# Low-cost feedback-controlled syringe pressure pumps for microfluidics applications

**DOI:** 10.1371/journal.pone.0175089

**Published:** 2017-04-03

**Authors:** John R. Lake, Keith C. Heyde, Warren C. Ruder

**Affiliations:** 1 Department of Bioengineering, University of Pittsburgh, Pittsburgh, PA, United States of America; 2 Department of Mechanical Engineering, Carnegie Mellon University, Pittsburgh, PA, United States of America; Northeastern University, UNITED STATES

## Abstract

Microfluidics are widely used in research ranging from bioengineering and biomedical disciplines to chemistry and nanotechnology. As such, there are a large number of options for the devices used to drive and control flow through microfluidic channels. Commercially available syringe pumps are probably the most commonly used instruments for this purpose, but are relatively high-cost and have inherent limitations due to their flow profiles when they are run open-loop. Here, we present a low-cost ($110) syringe pressure pump that uses feedback control to regulate the pressure into microfluidic chips. Using an open-source microcontroller board (Arduino), we demonstrate an easily operated and programmable syringe pump that can be run using either a PID or bang-bang control method. Through feedback control of the pressure at the inlets of two microfluidic geometries, we have shown stability of our device to within ±1% of the set point using a PID control method and within ±5% of the set point using a bang-bang control method with response times of less than 1 second. This device offers a low-cost option to drive and control well-regulated pressure-driven flow through microfluidic chips.

## Introduction

Microfluidic systems have become one of the more prolific tools for researchers in the chemical and biological sciences. The small volumes of reagents and samples required for use in microfluidic systems, combined with a widespread ability to fabricate high performance microfluidic chips using poly(dimethylsiloxane) (PDMS), makes microfluidics especially attractive for bioengineering and biomedical research [1–3]. As a platform, PDMS-based microfluidic devices are used for a wide range of applications including immunoassays, separation of proteins and DNA, and the sorting and manipulation of living cells allowing for researchers to gain insights into cell biology [4]. However, microfluidic systems are not limited to biological research, as they have been used by nanotechnology researchers as a high-throughput way of producing nanodevices [5] and as a platform for many analytical chemistry techniques such as electrophoresis and chromatography [6].

With such widespread use of microfluidic systems, there is a similarly broad range of methods to drive and control flows within these systems. The most well-characterized method for driving fluid through a microfluidic system is by using pressure-driven flow, due to its well-characterized flow profile [7, 8]. Some commonly used microfluidic flow control systems are peristaltic and recirculation pumps [9], pressure controller systems [10], and syringe pumps [11]. Peristaltic and recirculation pumps are easy to use, but have strong pulses in their flow due to their nature and are not very reliable. Pressure controller systems offer high performance and pulseless flows with fast responses, but are expensive and require an external pressure source. Syringe pumps are the most commonly used devices as they offer more stable flows than peristaltic or recirculation pumps, but are similarly easy to use and setup.

Due to the ubiquity of syringe pumps in laboratories performing microfluidic experiments, custom-built syringe pumps offer a low-cost option to expensive commercially available syringe pumps. Additionally, open-source syringe pumps have been developed which use 3D printed components to further reduce the cost and allow for these designs to be publically shared and widely used [12]. However, syringe pumps are typically operated open loop, specifying a particular rate at which the syringe is actuated, directly controlling the flow rate through the chip. While the mean flow rate for a period of time can be very accurate while using syringe pumps, the transient flow through the system is pulsed, with the pressure fluctuating over time within the chip. Additionally, syringe pumps have long response times, which limit their use in microfluidic studies requiring dynamic flow profiles [13, 14].

Here, we present a low-cost syringe pressure pump design that incorporates pressure feedback control to allow more responsive and stable control of flows through a microfluidic chip. In total, the system cost is approximately $110 for all required components (S1 Table). This approach offers an alternative to high cost commercial equivalents and is a more flexible solution for custom-built systems often used within a research laboratory setting. This syringe pressure pump is open-source from both a hardware and software perspective and takes advantage of 3D printing technologies to lower its cost and allow the designed components to be shared publically and built by any researcher. Additionally, we have developed two control methods for accurately controlling pressure-driven flows within microfluidics chips using a proportional, integral, and derivative error (PID) method and a bang-bang method.

## Results and discussion

### System design and characterization

**Fig 1:**
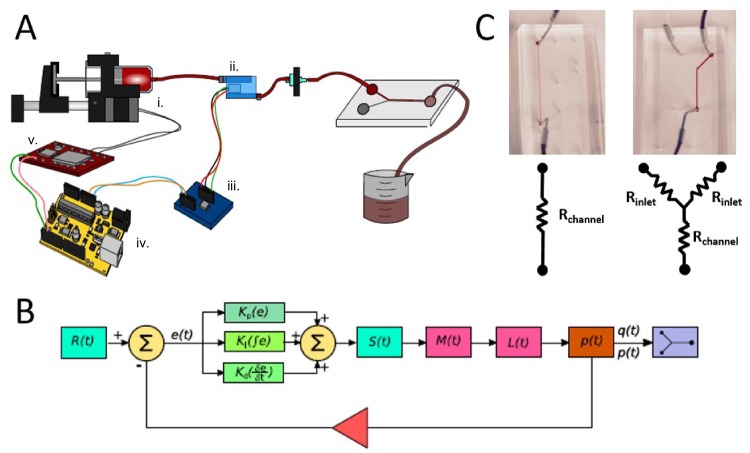
Syringe Pressure Pump Design Experimental Setup. A) An illustration of our syringe pump design. The liquid containing syringe is held in place by the 3D printed syringe pump parts, shown in black. These parts connect to a stepper motor (i.), depicted with grey and black stripes. When this stepper motor is actuated, the syringe pushes liquid through Tygon tubing, which passes through a piezoresistive pressure sensor (ii.) before entering a microfluidic channel. The electrical signal from the sensor is passed to an instrumentation amplifier (iii.), shown with the dark blue rectangle, before being transmitted and received by analog pins on an Arduino microcontroller (iv.). In response to these signals, the Arduino actuates the syringe pump via a stepper motor driver (v.), closing the feedback loop. B) Block diagram representation of the PID control structure detailing a mathematical abstraction for the feedback loop illustrated in Fig 1A. R(t) represents the commanded pressure, while e(t) represents the error in pressure between command and actual pressure, p(t). S(t) represents the step signal from the stepper driver. M(t) represents the stepper motor position, while L(t) represents the linear position of the syringe plunger that actuates flow through the syringe into the microfluidic system. q(t) is the flow rate through the system. C) Two different microfluidic chip geometries were used for this experiment, a linear channel shown on the left hand side of (C) and a Y-junction chip shown to its right. Both of these geometries may be modeled using hydraulic resistance abstractions for laminar flow regimes encountered within typical microfluidic testing conditions.

Our syringe pressure pump system is composed of four main components: the syringe pump, the pressure sensor and amplifier, the microcontroller, and the motor driver (Fig 1A). The syringe pump provides positive displacement that forces fluid through a microfluidic chip. It is designed from low-cost 3D printed acrylonitrile butadiene styrene (ABS) plastic parts and widely available, standard mechanical components. The pressure sensor and amplifier create an electrical signal, with voltage corresponding to the fluid pressure inside of the line, which is easily measured using an Arduino microcontroller board. When the microcontroller samples pressure signals from the sensor, it sends an output signal to the motor driver, actuating the syringe pump system, either increasing or decreasing the pressure within the fluid input line. Together, these components create an easily operated and programmable syringe pump with the advantages of a well-regulated pressure controller.

To control the pressure within the microfluidic chip, two control methods were employed. First, we implemented a bang-bang control method. This method was shown to be a reliable, yet extremely simple, way to maintain pressure set points in applications where high accuracy and fast response are not necessary. Similarly to the way many thermostats operate, the bang-bang control method operates by driving the system only when the pressure is outside a predetermined error threshold near a set point. In practice, this programming causes the bang-bang controller to drive the pressure in the line by actuating the syringe pump at a constant rate until the pressure exceeds a certain threshold from the set point. At that point, the controller stops driving the syringe pump, allowing for the pressure within the channel to decrease. This decline continues until the pressure is below the set point threshold, at which time the bang-bang controller will again drive the pressure, repeating this process to maintain a certain threshold of pressure. In addition, a higher performance PID controller (Fig 1B) was also implemented. This second strategy turned our syringe pump system into a highly responsive and stable pressure controller. However, unlike the plug-and-play bang-bang control method, this controller does require that the proportional, derivative and integral gains are tuned for the particular microfluidic chip design and application for optimal performance.

For characterizing the performance of our developed syringe pressure pumps, we used simple, well-characterized microfluidic geometries (Fig 1C). For measuring the response, we used a linear, rectangular channel of a constant height and width. For controlling kinematic boundary interactions between coflowing laminar interfaces driven by multiple syringe pressure pumps, we used a “Y-junction” geometry. The simple geometries of these chips allow us to use well-established microfluidic approximations [8, 15], enabling us to accurately characterize our pressure-driven flow while minimizing extraneous, and potentially confounding, variables.

### PID and bang-bang control responses

**Fig 2:**
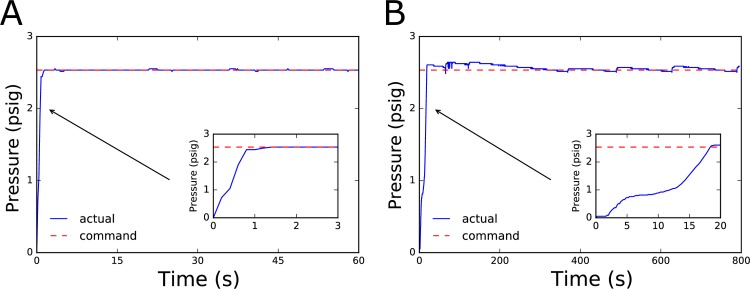
PID and Bang-bang Pressure Control Response Curves. A) Tuned PID controller response to a step command for a pressure set point of 2.5 psig. The response time is approximately 1 second with stability around the set point of ±1%. B) Bang-bang controller response where syringe pump is stepped at a constant speed to the set point. Response time is approximately 20 seconds with stability around the set point of ±5%. Both PID and bang-bang controller responses were measured using the linear microfluidic channel previously depicted.

To evaluate the effectiveness of our low-cost syringe pump system, we tested both the PID method and bang-bang control methods using well-characterized microfluidic chips (Fig 1C). Our findings indicated that the PID control method allows for a fast response time, approximately 1 second, to reach a desired set point while providing a high level of stability within ±1% of the set point over time (Fig 2A). Contrasted to the bang-bang controller, the PID method offers advantages in terms of a faster response and higher stability. However, the PID controller requires an additional application-specific parameter tuning process in order to achieve the highest performance. In contrast, the bang-bang control method is simple to implement and requires no tuning. It does have a slower response, of approximately 20 times that of the PID controller for our characteristic chip geometry tested, but allows for stability of ±5% which can be useful in many practical applications.

Aside from performance, the choice between these two control methods has implications for power utilization and the ability for these pumps to be mobile. As the PID control method is always actively driving the system to maintain the set point, it requires that the motor driver is always enabled, energizing the windings of the stepper motor that actuates the syringe pump. However, the bang-bang control method is only actively driving the system towards the set point for a fraction of the time when the pressure has fallen below the threshold of the pressure set point. For this reason, any time the pressure is above or within the threshold of the set point, the stepper motor driver can be disabled, and the windings of the stepper motor can be de-energized. As the typical pressures involved in driving flows through a microfluidic chip are relatively low, the threaded rod itself is capable of keeping the syringe engaged without risk of backlash or undesired movement during these periods. For most laboratory settings, the high current draw by the stepper motor within a syringe pump does not limit its use. However, for battery-powered systems, the large current draw by stepper motors limits their potential for mobile field deployment. Combining the capability for the developed syringe pressure pumps to operate in a low-power bang-bang control mode with their small size and weight relative to traditional syringe pumps, makes them viable flow controllers for applications within mobile or field-deployed systems.

### Programmable profiles using PID controller

**Fig 3:**
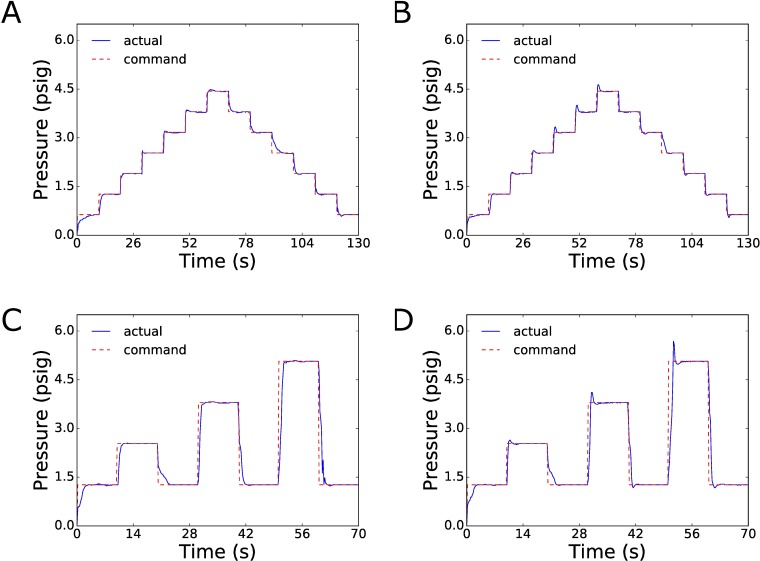
Step and Pulse Pressure Profile Responses. A) Tuned PID response profile to increasing and decreasing step commands, with each pressure step of 0.63 psig. B) Tuned PI response profile to increasing and decreasing step commands, with each pressure step of 0.63 psig. C) Tuned PID controller response profile to increasing pulsed pressure commands, with each pressure pulse doubling, tripling and then quadrupling the original pressure set point of 1.25 psi. The derivative gain may be increased and tuned to assure that little or no overshoot occurs. D) Tuned PI controller response profile to increasing pulsed pressure commands, with each pressure pulse doubling, tripling and then quadrupling the original pressure set point of 1.25 psi. All pressure profiles were obtained using the linear microfluidic chip geometry.

The developed syringe pump is inherently programmable through the Arduino integrated development environment. To verify our controllers, we tested a stepwise pressure profile and a pulsed pressure profile (Fig 3). Having found that our bang-bang method is not well-suited for applications requiring frequent or rapid changes in the pressure set point over time, we focused on testing the PID control method for dynamic pressure profiles. Accordingly, we employed the PID control method for testing our syringe pressure pumps to follow the stepwise and pulsed pressure profiles. During testing, the syringe pressure pumps were able to accurately follow their commanded pressure profiles (Fig 3). Additionally, the PID tuning process can be used to optimize the pressure response for these pumps to suit a specific application. In many biological applications, pressure overshoot can cause issues and would therefore need to be minimized. In these cases, the derivative gain for the controller can be increased to ensure that little to no overshoot occurs (Fig 3A and 3C). Alternatively, if a fast pressure response is required and overshoot is allowable for a given application, the controller’s derivative component can be removed, creating a PI controller to decrease the response time for the system (Fig 3B, Fig 3D).

These pressure profiles are used to illustrate the flexibility of the developed syringe pressure pumps due to their pressure feedback control. As the pressure profiles can be customized by the user, the pumps offer the ability to control pressures to suit a specific microfluidic application. Also, due to the way that our syringe pumps were developed, they are easy to integrate with other systems, making them a flexible component that can be incorporated into custom lab setups.

### Laminar interface position control and stability

Being able to actively move and control the relative interface position of laminar, coflowing streams within microfluidic channels is useful [16]. Due to the laminar characteristics of flows within microfluidic systems, no turbulent mixing occurs and the adjacent flow streams remain separated by a defined kinematic boundary. Controlling this interface has been used for subcellular domain labeling and stimulation [17], for cell patterning [18], and for microfabrication within microfluidic chips [19].

**Fig 4:**
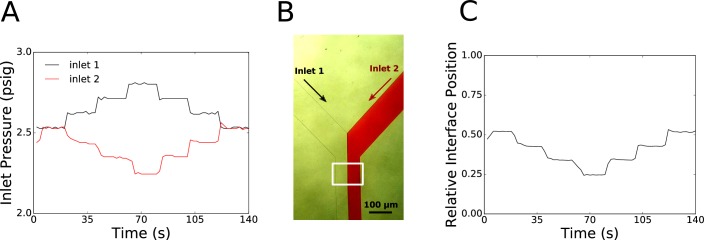
Laminar Interface Position Control. A) Tuned PID controller maintained a constant total pressure at both inlets of 5 psig, while adjusting the pressures at the inlets to move the relative interface position within the Y-junction microfluidic chip. B) An illustration of the images captured during the interface control experiment showing the two inlets on the Y-junction chip as well as their downstream convergence. Inlet 1 contains deionized water, while inlet 2 contains red food coloring to act as a dye. Image captured using a 40x stereomicroscope directly using a Raspberry Pi camera module v1.3. The image was contrast enhanced 0.5% and sharpened using ImageJ software. C) The plotted interface position as a function of time showing how the interface position can be controlled using the syringe pressure pumps.

We demonstrated the versatility of our syringe pump system by controlling and modulating the pressure at two inlets of a Y-junction microfluidic chip and optically measured the resulting interface position. As shown in Fig 4, the relative interface position was able to be controlled in a stable fashion by adjusting the pressure set points at the inlets of the Y-junction chip using our syringe pressure pumps. Images were captured and post-processed to determine the interface position at each data point where pressure measurements were recorded.

To compare the interface stability of open loop syringe pumps with our syringe pressure pumps, we measured the pressure of the two inlets of our Y-junction microfluidic chip and simultaneously captured images of the interface position for three different scenarios. The first scenario involved a single commercial syringe pump (PHD 2000, Harvard Apparatus) operating both syringe pumps for the two inlets to the chip. The second scenario involved using two of our developed syringe pumps independently operating syringes to the two inlets of the chip running at the same constant rate. Finally, the third scenario used these same syringe pumps, but incorporated pressure feedback control to hold both inlets at the same pressure set point using the PID control method.

**Fig 5:**
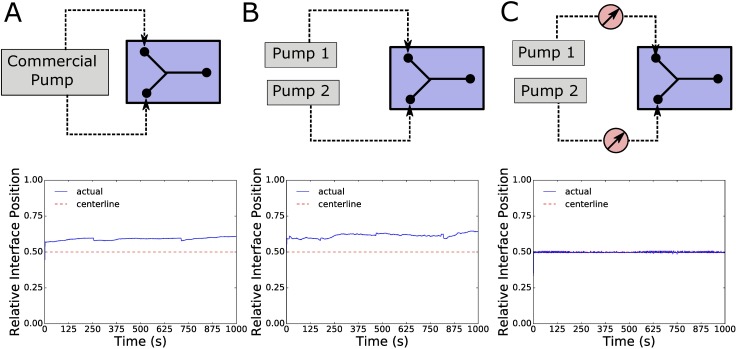
Laminar Interface Stability Comparison. A) The first scenario used a single commercially available syringe pump (Harvard Apparatus PHD2000, Model #HA2000W) running open loop at a constant flowrate, operating both syringes connected to the inlets of the Y-junction chip. B) The second scenario used two separate, custom made syringe pumps running open loop at the same constant flow rate, operating the syringes connected to the inlets of the Y-junction chip independently. C) The third scenario used two separate, custom made syringe pressure pumps using feedback pressure control to maintain equal pressures at both inlets to the Y-junction chip.

For the first scenario (Fig 5A), although a single commercial syringe pump was driving the flow at a constant flow rate for both inlets to the Y-junction, the interface position had periodic fluctuations, characterized by a slow drift with a period rapid displacement. Additionally, the interface position was not centered in the channel, as we would expect if the flows were moving with the same flow rate. We observed similar behavior of the interface position when using two of our syringe pumps to operate the inlet syringes independently in an open loop fashion (Fig 5B). However, once the pressure feedback is added to the same setup to maintain equivalent pressure at the inlets (Fig 5C), the interface position is much more stable over time, without significant fluctuation or drift and also is closely positioned at the center of the channel as we would expect.

As shown in Fig 5, syringe pumps running open loop are not reliably controlled to allow for predictable interface positions, as they fluctuate and drift over time. The stability of multiple coflowing reagents into microfluidic channels is important in many cases. such as generating temporal or spatial concentration gradients within the channel for experimental purposes [20] or for the generation of droplets [21]. Especially for long-term experiments or those involving sensitive biological specimen, this lack of control over the pressures within the channel by using syringe pumps in an open loop fashion can affect researchers’ ability to properly control and predict the flow within their microfluidic devices.

## Materials and methods

### Design and fabrication of microfluidic chips

Designs of the microfluidic channels were created in a CAD program (AutoCAD 2016, Autodesk). High resolution (20000 dpi) transparencies were then produced from the created CAD drawing files (CAD Art Services Inc.). These transparencies were then used as masks in photolithography on negative photoresist spin coated onto silicon wafers to create master wafers for our microfluidic chip designs. After development of the photoresist, the masters were placed into a vacuum chamber overnight with 50 μL of tridecafluorooctyltrichlorosilane for silanization of the master wafer’s surface which aids in the release of PDMS from the master after molding. A mixture of 10:1 PDMS prepolymer and curing agent (Sylgard 184, Dow Corning) were well mixed together and then degassed in a vacuum chamber. The degassed mixture was then poured onto the master for molding. The master was then placed into an oven at 80°C to expedite the curing process. After fully curing, the PDMS was removed from the master mold and cut into individual microfluidic chips. Next, inlet and outlet holes were punched into the individual chips using a blunt tipped stainless steel dispensing needle (Stainless Steel Dispensing Needle Straight, 23 Gauge, Catalog # 75165A684, McMaster Carr Supply Company). Finally, each PDMS microfluidic chip was bonded to a glass cover slide (40 x 22 mm) by conformal contact after both the PDMS chip and glass cover slide were oxidized in a plasma cleaner (Plasma Cleaner PDC-32G, Harrick Plasma) for 1 minute. After the PDMS chip is bonded to the glass cover slide, it is placed in an oven at 80°C for an additional two hours to ensure a tight bond.

### Pressure sensor calibration and signal conditioning

In-line piezoresistive pressure sensors (PendoTECH Single Use Pressure Sensor PRESS-S-000) were used for each syringe pressure pump. Although these sensors are priced for single-use, they can be sterilized by washing with a 70% ethanol solution and reused many times, offering a low-cost sensor that is high accuracy in a small and convenient package for this application. To calibrate the sensors, we applied known pressures to the inlet of the sensor, with the outlet capped, and measured the resulting output signal; see the calibration curves included in the supporting information section for additional details (S1 Fig). For signal conditioning, we used a single supply, micropower instrumentation amplifier (Texas Instruments INA122P) having a typical supply voltage of 10 V. For these experiments, we utilized an amplifier gain of approximately 435. As the Arduino board’s logic level is 5 V and the instrumentation was being powered with 10 V, we used a simple voltage divider circuit to safe-guard the output signal to be no more than 5 V. For the full circuit diagram, refer to S2 Fig.

### Data acquisition and analysis

#### Pressure signal acquisition

Pressure signals were acquired using an Arduino Uno Rev 3 microcontroller board which uses an ATmega328P microcontroller. The Arduino board includes analog pins with 10-bit resolution which can be used to measure analog voltage signals to be used directly by the microcontroller. Using the Arduino’s analog pins, the conditioned analog signals from the pressure sensor were read by the Arduino every 200 milliseconds and used by the microcontroller to actuate the stepper motors accordingly to maintain pressure set points. Data was transferred directly to a laptop computer using a serial interface from the Arduino via a USB connection. The data was transferred at a baud rate of 9600 bits per second and the pressure sensor’s signal along with the time elapsed was collected for each sensor measurement.

#### Image capture of laminar interface position

For image capture of the interface position, we connected an Arduino board to an open-source microcomputer (Raspberry Pi 2 model B) via a serial interface so that pressure measurements taken by the Arduino could trigger an image to be taken by the microcomputer. To take magnified images, we connected a camera module made for the microcomputer (Raspberry Pi camera module v1.3) to one of the eyepieces of a stereomicroscope with 40x magnification (Carolina Biological Supply, Catalog #591846). Using a Python program, we took images at a prescribed interval as the Arduino collected pressure measurements. These images were used to determine the interface position by counting the pixels that were dyed relative to the pixel count for a given region slightly downstream of the confluence of the Y junction inlets. Processing of these images was done within MATLAB R2015B.

### Syringe pressure pump design and 3d printing

Syringe pump design was inspired by open-source designs previously published and either used directly or modified to suit our applications for this syringe pressure pump [12]. The syringe pressure pumps were constructed from a combination of custom 3D printed components made from acrylonitrile butadiene styrene (ABS) plastic along with standard mechanical hardware. The 3D printed parts were designed using a CAD software program (Inventor 2017, Autodesk) and 3D models were exported as STL files. These STL files were then opened in a software package (Z-Suite, Zortrax) dedicated to the conversion of STL models to the executable instruction set, its equivalent gcode, for the Zortrax family of 3D printers. These parts were then printed using a commercially available fused deposition modeling 3D printer (Zortrax M200, Zortrax). As STL files are a standard format for 3D models and used extensively for 3D printing platforms, the models included as supporting information to this paper can be downloaded and used across other 3D printing software and hardware. Upon printing, the parts were then assembled with widely available, standard mechanical parts, including machine screws, threaded rod, linear and ball bearings, linear shafts and couplings. For a listing of the standard mechanical parts used, refer to the bill of materials for the pump which can be found in S1 Table.

## Conclusions

The syringe pressure pump demonstrated here provides a flexible tool for researchers that can be used in a variety of applications requiring well-regulated pressure-driven flow for microfluidics. These pumps can offer an easy-to-use device which can be fabricated in less than one day at a very low cost. It also can be used across many different research areas, as pressure-driven microfluidics spans a broad range of disciplines. This syringe pressure pump demonstrates that relatively high performance flow control can be achieved by combining low-cost hardware and electronics with fundamental control algorithms. Additionally, with the continuing advancement of 3D printing technologies, academic labs are increasingly relying on rapid prototyping techniques to accelerate the development of tools used in their research. For this reason, these pumps can provide a broad audience of researchers access to a useful tool for microfluidics studies.

## Supporting information

S1 Fig Calibration Curves for the Pressure Sensors Used. These curves show calibration curves of the sensors that were used during experimentation. An amplifier gain of 611 was used for calibration of sensors. Each sensor’s calibration data was fitted to a linear regression model using the least squares method. The coefficient of determination, or R^2^ value, is determined by subtracting the ratio of the residual sum of squares by the total sum of squares from 1. The residual and total sum of squares are defined by the following equations respectively, ∑_i_ (*y*_*i*_*—y*_*mean*_)^2^ and ∑_i_ (*y*_*i*_−*f*(*x*_*i*_))^2^.
(TIF)

S2 Fig Microcontroller and Pressure Sensor Circuit Diagram. This is a circuit schematic (created using Fritzing, under GNU GPL v3 license) that shows the components and connections used to allow the Arduino microcontroller board to measure amplified pressure signals from the pressure sensor used in the syringe pressure pump’s design.
(TIF)

S1 File Syringe Pump Component STL Files. Zipped folder containing all the required STL files to 3D print the required ABS components for the syringe pressure pump presented here.
(ZIP)

S2 File Syringe Pump Arduino Control Programs. Zipped folder containing the Arduino sketches required to control both the Bang-bang and PID control methods presented here. For the PID control method, 2 Arduino sketches are included; one to control a single pressure pump and another to control two pressure pumps simultaneously.
(ZIP)

S1 Table Bill of Materials Listing. Bill of materials listing the parts, their quantity, approximate unit and extended costs to create the designed syringe pressure pump.
(TIF)
