# Supplementary figures and images for: Low-cost feedback-controlled syringe pressure pumps for microfluidics applications
Source: PLoS One. 2017 Apr 3;12(4):e0175089. doi: 10.1371/journal.pone.0175089 (PMC5378403; doi:10.1371/journal.pone.0175089)

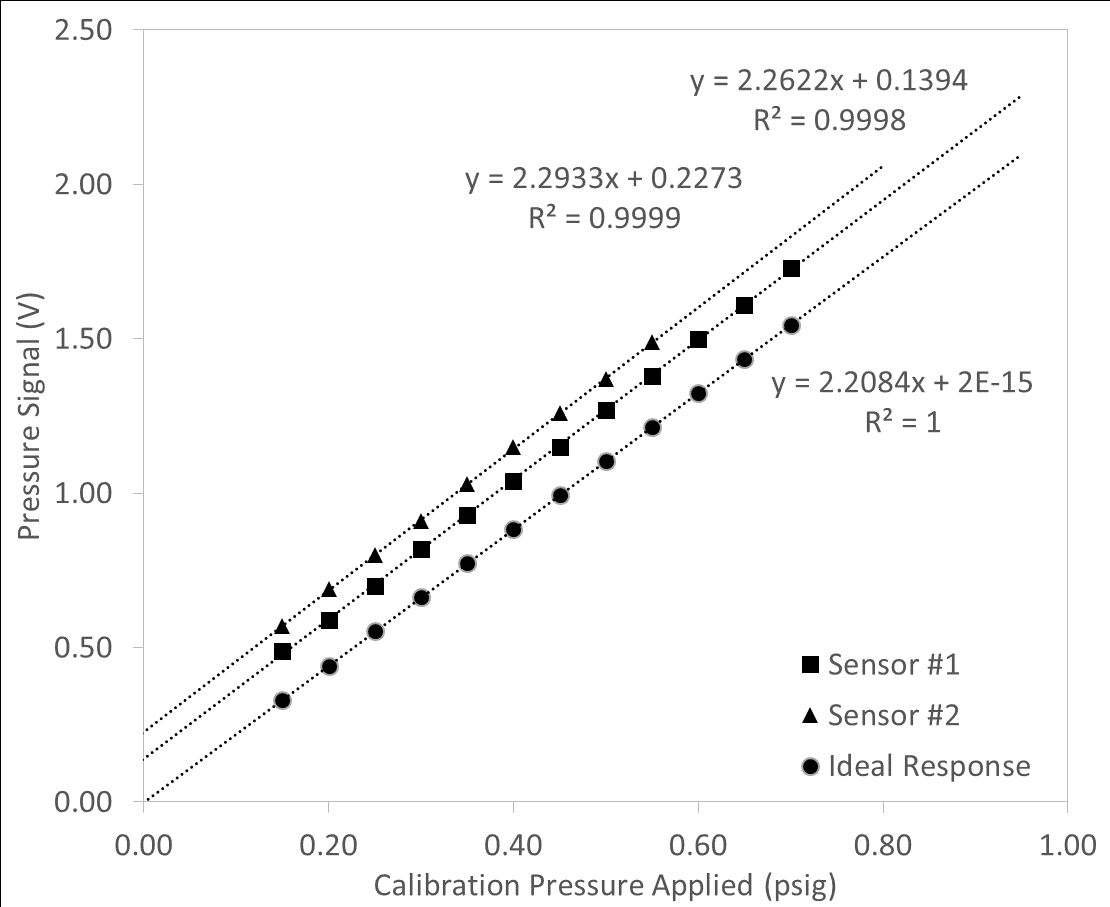

Supplement: S1 Fig — These curves show calibration curves of the sensors that were used during experimentation. An amplifier gain of 611 was used for calibration of sensors. Each sensor’s calibration data was fitted to a linear regression model using the least squares method. The coefficient of determination, or R2 value, is determined by subtracting the ratio of the residual sum of squares by the total sum of squares from 1. The residual and total sum of squares are defined by the following equations respectively, ∑i (yi—ymean)2 and ∑i (yi−f(xi))2. (TIF) [file pone.0175089.s001.tif]

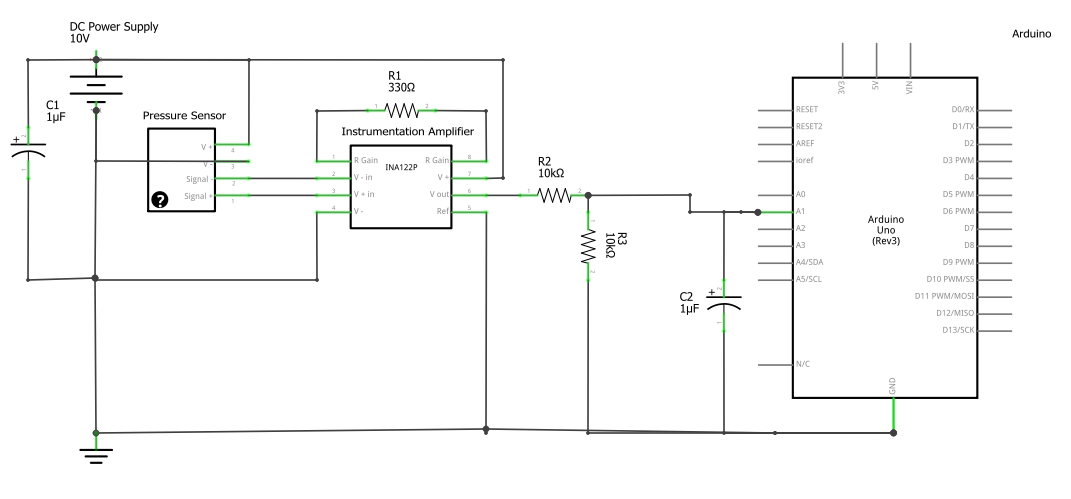

Supplement: S2 Fig — This is a circuit schematic (created using Fritzing, under GNU GPL v3 license) that shows the components and connections used to allow the Arduino microcontroller board to measure amplified pressure signals from the pressure sensor used in the syringe pressure pump’s design. (TIF) [file pone.0175089.s002.tif]

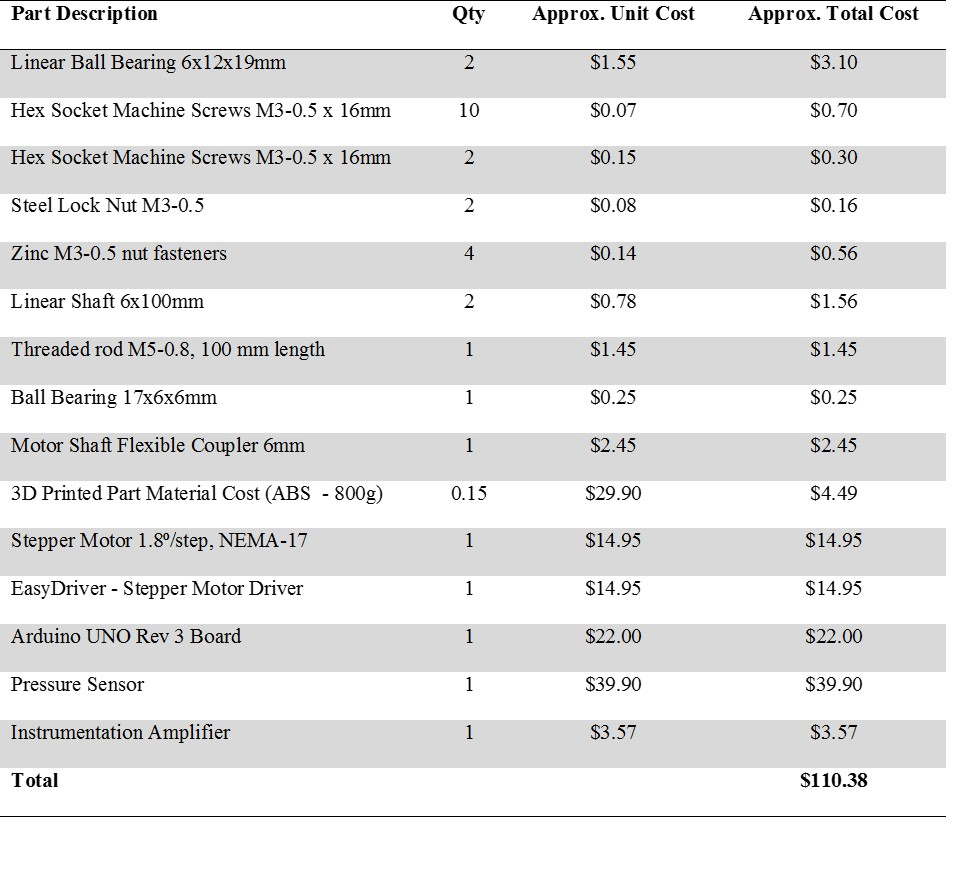

Supplement: S1 Table — Bill of materials listing the parts, their quantity, approximate unit and extended costs to create the designed syringe pressure pump. (TIF) [file pone.0175089.s005.tif]
